# Does speech rate influence intertemporal decisions? an experimental investigation

**DOI:** 10.1371/journal.pone.0264356

**Published:** 2022-02-25

**Authors:** Josie I. Chen, Tai-Sen He, Hsin-Ya Liao

**Affiliations:** 1 Department of Economics, National Taiwan University, Taipei City, Taiwan; 2 Economics Programme, School of Social Sciences, Nanyang Technological University, Singapore, Singapore; Texas A&M University, UNITED STATES

## Abstract

This paper experimentally examines the effect of speech rate on intertemporal decisions. In a delay-discounting task, subjects made a series of intertemporal choices between smaller-sooner and larger-delayed rewards and were asked to listen to a voice recording verbalizing the information for payoff options. We manipulated the speech rate of the voice recordings and administered two treatment conditions: *Slow* and *Fast*. We did not find an overall treatment effect in the acoustic manipulation.

## 1. Introduction

Many economic transactions are facilitated through certain forms of verbal communication. Although standard economic models focus primarily on payoff-related information, nonverbal cues also meaningfully shape individual perception and judgment. For example, speech rate, the pace at which people speak, is a salient nonverbal element in communication. Existing psychological studies have shown that speech rate influences listeners’ perception and judgment of a speaker’s personality attributes [1–5]. Yet, little is known about how speech rate affects listeners’ subsequent decision-making, especially in economically important contexts. The present study thus aims to examine the speed rate effect on people’s intertemporal decisions in a controlled experiment.

Our main research hypothesis builds on the dual-systems theories [6, 7]. Such theories postulate that the hot system tends to be triggered by options with immediate rewards, leading to myopic and impulsive choices, whereas the cool system deliberates the relative valuation of immediate and delayed rewards and then analyzes outcomes in a future-oriented perspective. Prior research has shown that listening to fast speech can lead to a faster rate in subsequent tasks [8–11]. Notably, Buelow et al. [8] showed that speech rate primes decision-making speed given that, in their findings, faster speech resulted in faster decisions and vice versa. Thus, according to the dual-systems framework, if a faster speech rate causes faster decisions, the hot system would induce fewer future-oriented decisions. A slower speech rate would, however, lead to slower decisions. Under such circumstances, individuals would rely primarily on the cool system and, subsequently, behave more patiently.

The predominant view in psychological and economic models based on the dual-systems theory supports that increased cognitive load induces intuition but inhibits deliberation, leading to more impulsive decisions. Empirical evidence, however, is rather mixed. Earlier studies, such as Hinson et al. [12] and Shiv and Fedorikhin [13], found that subjects behaved more impatiently when a cognitive load (e.g., memorizing a 5- or 7-digit number) was imposed during the intertemporal decision-making process, compared to those without an imposed cognitive load. More recently, several studies reported that taxing cognitive resources does not necessarily increase impulsive decision-making [14–17]. In particular, Olschewskim, Rieskamp, and Scheibehenne [17] demonstrated that taxing cognitive resources does not alter preference but instead reduces choice inconsistency. In the present study, we test the main research hypothesis that a slower speech rate facilitates patience against the null hypothesis that the speech rate does not change intertemporal decisions.

## 2. Methods: Experiment design and procedures

We elicited intertemporal preferences using a modified version of the multiple-price-list method [18, 19]. The decision-making task comprised 24 rounds. In each round, subjects made binary choices between NTD $100 paid today and a larger amount paid in m weeks, with m equal to 1, 4, and 12 weeks to indicate the duration of delay. The larger delayed monetary amount took on 8 different values: $105, $110, $115, ……, $135, and $140. The decision items were presented to the subjects in random order. The order of m was randomized, while the delayed amount was presented to the subjects in an increasing order, starting at $105 and increasing in $5 increments. The payoff information was conveyed to the subjects through audio clips in which a Google automated attendant verbalized the options. Subjects were asked to listen to the audio clip before making a decision.

Our acoustic manipulations were naturally embedded in the voice recordings. We used speech synthesizer to generate two speech rate conditions: *Fast* and *Slow*. The duration of the voice recording verbalizing a decision option (“You will receive m dollars in n weeks.”) was 2.8 seconds in the *Fast* condition and 3.1 seconds in the *Slow* condition. The subtle change in speed rate ensures the voices in both versions were clear and natural-sounding to the subjects, avoiding potential confusion and demand effects. In fact, we included a control question asking subjects to guess the purpose of the study in the post-experiment questionnaire. No subjects correctly answered this question. We employed the between-subject design. Each subject participated in the experiment only once and was randomly assigned to the *Fast* or *Slow* condition. Additionally, two female experimenters, blind to our research hypothesis and the ongoing treatment condition, administered all sessions. Randomization was implemented through a pre-programmed random number generator.

We aimed to collect an effective sample size of 100 observations per condition. Our power calculation was based on Chen and He’s [19] recent study that used a similar delay-discounting task to examine a subtle linguistic intervention and observed an effect size of roughly 0.5 standard deviation. Conservatively, we anticipated that the speech rate intervention would generate an effect size of 0.4 standard deviation. The selected sample size of 100 per condition is large enough to detect this effect size at a 5% significance level with statistical power of 80%. In fact, our sample size was no smaller than that in Chen, He, and Riyanto’s [20] study, which tested a subtle one-word intervention, adopted a multiple-price-list method to elicit time preference, and reported a null result.

The study was approved by the National Taiwan University Institutional Review Board, and all participants provided informed consent in a written form. We conducted the experiment with a total of 224 student subjects at National Taiwan University; subjects were recruited using the Taiwan Social Sciences Experimental Laboratory (TASSEL) recruitment system. All sessions were conducted in Mandarin Chinese and administered in an individual format with only one subject in each session, lasting for roughly 20 minutes. After the instructions stage, subjects made 24 binary decisions in the time preference elicitation task and then completed a post-experiment questionnaire (please see S1 Appendix for details). Payments were made privately at the end of the session. The average earnings were NTD$218 (roughly equivalent to US$7). Subjects’ earnings comprised a guaranteed participation fee and an incentive payment, based on a randomly chosen round in the delay-discounting task. The participation fee of NTD$100 was paid in cash on-site, while the incentive payment was paid in the form of a wire transfer to minimize the possible differences in transaction costs and payment risks associated with different payment timing. The experiment was programmed and conducted using oTree [21].

## 3. Results

A total of 224 subjects participated and successfully completed the experiment. Table 1 provides summary statistics for the participants. Nearly half (43%) were female, about a quarter (27%) were economic or business majors, nearly all (95%) were local students, and more than three quarters (77%) were undergraduate students. We performed pairwise proportion tests to test the difference of the means and found no significant differences between the two conditions for any of the demographic variables, suggesting that the random assignment is valid.

**Table 1:** Descriptive statistics of demographic variables.

	*Slow*	*Fast*	All Subjects
*Female*	0.46	0.40	0.43
	(0.50)	(0.49)	(0.50)
*EconMajor*	0.31	0.23	0.27
	(0.46)	(0.42)	(0.44)
*Local*	0.96	0.95	0.95
	(0.20)	(0.22)	(0.21)
*Undergraduate*	0.76	0.79	0.77
	(0.43)	(0.41)	(0.42)
No. of obs.	98	100	198

Note: Observations exhibiting inconsistent decisions are excluded. Standard deviations are reported in parentheses. Pairwise proportion tests were used to test the difference of means. None of the means are significantly different at the 5% level. *Female* is a dummy that equals 1 if the subject is female and 0 otherwise. *EconMajor* is a dummy that equals 1 if the subject is an economics or business-related major and 0 otherwise. *Local* is a dummy that equals 1 if the subject is a local student and 0 otherwise. *Undergraduate* is a dummy that equals 1 if the subject is an undergraduate student and 0 otherwise.

After excluding inconsistent decisions with multiple switch points, we had 198 valid observations for data analysis. Table 2 shows, by treatment, the average minimum amount that subjects required to switch from the immediate to the delayed option (i.e., the switch point). Pooling observations from the two conditions, subjects required, on average, a payment of $112 in 1 week, $119 in 4 weeks, and $126 in 12 weeks to switch from the NTD$100 immediate payment. Subjects in both conditions unanimously required a higher reward amount for a longer delay duration, indicating that people discounted the delayed rewards in the distant future more heavily than those in the near future. We do not observe an overall treatment effect of speech rate. Using the two-sided Mann–Whitney test, the switch point is not statistically different between the two conditions for each duration of delay (1, 4, and 12 weeks) and aggregate level (i.e., mean).

**Table 2:** Comparison of switch points by treatment.

Treatment	*Slow*	*Fast*	All Subjects
1 week	111.68	112.10	111.89
	(10.48)	(11.06)	(10.75)
4 weeks	117.70	120.05	118.89
	(13.96)	(14.03)	(14.01)
12 weeks	124.54	127.60	126.09
	(15.10)	(15.41)	(15.30)
Mean	117.98	119.92	118.96
	(12.22)	(12.36)	(12.30)
No. of obs.	98	100	198

Note: Switch point is the minimum amount a subject required to switch from the immediate option to the delayed option. Standard deviations are reported in parentheses. 1 week, 4 weeks, and 12 weeks are the durations of delay.

As the subjects made 24 binary choices between an immediate and a delayed option, we performed probit regressions to estimate the treatment effect while controlling for a set of demographic variables, as shown in Table 3. Standard errors are clustered at the individual level, and the reported results are the average marginal effects. The dependent variable is a binary variable that equals 1 if the subject chose the delayed option and 0 if the subject chose the immediate reward. In Column 1, we regressed only the treatment dummy. *Slow* indicates the treatment dummy equals 1 if the subject is in the *Slow* condition; otherwise, the dummy takes the value of 0. Corresponding to the findings in Table 2, the coefficient of *Slow* is positive but not significant, indicating that subjects in the *Slow* condition did not discount the delayed option less heavily than subjects in the *Fast* condition.

**Table 3:** Regression results.

	All subjects		Male subjects		Female subjects	
	(1)	(2)	(3)	(4)	(5)	(6)
*Slow*	0.049	0.058	0.006	0.009	0.102	0.134*
	(0.044)	(0.052)	(0.058)	(0.069)	(0.068)	(0.081)
*Delay in weeks*		-0.034***		-0.037***		-0.032***
		(0.003)		(0.003)		(0.003)
*Reward amount*		0.017***		0.017***		0.016***
		(0.001)		(0.001)		(0.001)
*Female*		0.028				
		(0.593)				
*EconMajor*		-0.010		0.033		-0.102
		(0.060)		(0.076)		(0.101)
*Local*		0.375***		0.528***		0.210
		(0.108)		(0.103)		(0.148)
*Undergraduate*		0.118*		0.152*		0.096
		(0.066)		(0.085)		(0.103)
No. of obs.	4752	4752	2712	2712	2040	2040
No. of clusters	198	198	113	113	85	85

Note: Probit estimation. Reported results are average marginal effects. Robust standard errors corrected for clustering on the individual level are in parentheses. The dependent variable is a dummy that equals 1 if the subject chose the delayed option and 0 otherwise. *Slow* is the treatment dummy that equals 1 if the subject is in the *Slow* condition and 0 otherwise. *Delay in weeks* indicates the duration of delay in weeks. *Gained amount* is the amount of money the subject is paid. *Female* is a dummy that equals 1 if the subject is female and 0 otherwise. *EconMajor* is a dummy that equals 1 if the subject is an economics or business-related major and 0 otherwise. *Local* is a dummy that equals 1 if the subject is a local student and 0 otherwise. *Undergraduate* is a dummy that equals 1 if the subject is an undergraduate student and 0 otherwise.

*** p < 0.01

** p < 0.05, and

* p < 0.1.

In Column (2), we further controlled for the duration of delays (*Delay in weeks*), the amount of the delayed option specified in a round (*Reward amount*), and a set of demographic variables, including *Female* (*Female* = 1 if the subject is female; otherwise, *Female* = 0), *EconMajor* (*EconMajor* = 1 if the subject is an economics or business major; otherwise, *EconMajor* = 0), *Local* (*Local* = 1 if the subject is a local Taiwanese student; otherwise, *Local* = 0), and *Undergraduate* (*Undergraduate* = 1 if the subject is an undergraduate student; otherwise, *Undergraduate* = 0). Since people typically more heavily discount the delayed options when the wait for receiving the rewards is longer, we predict a negative relationship between *Delay in weeks* and the dependent variable. In addition, people are more inclined to choose the delayed option when the incentive size is increased. Thus, the coefficient of *Reward amount* is predicted to be positive. As expected, we observe a negative and significant coefficient of *Delay in weeks* and a positive and significant coefficient of *Reward amount*. The coefficients of *Female* and *EconMajor* are insignificant. The coefficients of *Local* and *Undergraduate* are positive and significant, indicating that local students and undergraduate students exhibited a higher level of patience than their corresponding counterparts. After the inclusion of these control variables, the coefficient of *Slow* remains not significant. Overall, we do not find an overall speech rate effect on intertemporal decisions.

We additionally performed a heterogenous analysis on gender. Much research has shown that women perform better than men on tasks measuring interpersonal sensitivity (e.g., Hall and Mast [22]), and we conjecture that the female subjects could be more sensitive to our subtle speech rate manipulation. We divided the full sample into two sub-groups (male and female) and conducted the same regression analysis. Columns (3)–(4) in Table 3 report the results from male subjects, and Columns (5)–(6) report the results from female subjects. The coefficients of *Slow* are very close to zero in Columns (3)–(4) but are positive in Columns (5)–(6) (p-value is slightly above 0.1 in Column (5) and under 0.1 in Column (6)). We further added the interaction term between *Slow* and *Female* to Column (2) to test whether the treatment effect was more pronounced among the female subjects than among their male counterparts. However, the coefficient of the interaction term is not statistically significant. Overall, our results show that the subtle speech rate manipulation somewhat influences female subjects’ intertemporal decisions but has nearly zero impact on male subjects’ decisions, albeit the difference is not statistically significant. Similar to our results, Rhodes et al. [23] found that subtle linguistic cues, such as describing science in terms of actions (let’s do science) versus identities (let’s be scientists), reduced the gender disparities in science engagement. He, Li, and Yan [24] also found that females were so much more responsive than males to subtle linguistic cues that the gender difference in risk aversion was closed when payoff information was described without the use of the first-person pronoun “I.”.

## 4. Discussion and conclusion

In many economic contexts, speech rate and other nonverbal cues play an important role in shaping individual judgements and decision-making. The present study examines the effect of speech rate on intertemporal decisions in a laboratory setting. We subtly embedded the speech rate manipulation in voice recordings that verbalize the payoff information for the decision options and then administered two treatment conditions: *Slow* and *Fast*. We did not find an overall treatment effect of speech rate. Interestingly, we observed a small gender difference in subjects’ responses to the acoustic manipulation. Our speech rate manipulation seemed to influence female subjects’ intertemporal decisions but had no impact on male subjects’ decisions.

Notably, however, there is a limitation of the present study. To ensure voice recordings sounded clear and natural to subjects, the speech rate differed only slightly between the *Fast* and *Slow* conditions in our experiment. Thus, a more intense manipulation may be required to observe an effect, highlighting an area worth further exploration.

Our study ultimately contributes to the literature in three ways. First, our study relates to the broader literature investigating how vocal characteristics influence people’s perceptions and decisions. To the best of our knowledge, our study is the first economics research to explore how speech rate affects intertemporal decisions in a controlled laboratory environment. Second, while psychological and economic models in dual-systems theory imply that the hot system induced by a faster speech rate would lead to more impatient decisions, we do not find supporting evidence of this effect. Instead, our findings align with several recent studies showing that increasing cognitive load does not necessarily alter intertemporal preference. Finally, our results are consistent with prior research showing that women are more responsive than men to nonverbal signals. We specifically highlight how a subtle drop in speech rate increases female subjects’ patience. Therefore, if aiming to influence people’s intertemporal decisions, policymakers or practitioners could achieve such influence simply by changing the pace at which they speak—keeping in mind, however, that only women will be “listening.”

## Supporting information

S1 Appendix Experimental instructions and questionnaire. (DOCX)
