# Supplementary material for: Does speech rate influence intertemporal decisions? an experimental investigation
Source: PLoS One. 2022 Feb 25;17(2):e0264356. doi: 10.1371/journal.pone.0264356 (PMC8880867; doi:10.1371/journal.pone.0264356)
Supplement: S1 Appendix — (DOCX) [file pone.0264356.s001.docx]

**S1 Online Appendix. Experimental Instructions and Questionnaire**

**A. Instructions, main task, and exit survey (English-translated version)**

**A.1 Experimental instructions**

Thanks for your participation. This session consists of a main task and a post-experiment questionnaire. I expect the whole session to take about 30 minutes. Depending on your decisions and luck, you will be able to earn a sum of money in addition to the $100 guaranteed for your participation. Please read the following instructions carefully.

If you have any questions, please raise your hand and the experimenter will come to assist you. Now please turn off your mobile phones and any other electronic devices. These must remain off until you leave this room.

Your earnings will be in New Taiwanese dollars in this experiment.

To ensure anonymity, your decisions in this session are only linked to your Participant ID number and at the end of this session you will be paid by Participant ID number. We will collect your name for audit purposes only. Subsequent researchers will not be able to link your name to your Participant ID. Your decisions in the experiment are confidential.

Next

**Main Task**

This task consists of 24 rounds. In each round, you will be choosing between “receiving 100 dollars today” and “receiving y dollars in x weeks” where x is positive, and y is greater than 100. That is, you will decide whether you are willing to wait for a couple of weeks to receive an amount larger than 100 dollars. Note that x and y will be assigned different values in every round. Please read and listen carefully.

Please put on the earphones and proceed to the next page.

 Next

**Example**

We will provide you an example before the real rounds start.

Please click “Next Page” to play the audio recording.

(The content of the audio recording is “I will receive 120 dollars in 2 weeks ”)

Note: this is an example for illustration purpose. The amount and date will be different in the real rounds.

(After Audio recording is played, the text below will be shown on screen)

Please choose if you would like to receive the payment today or in the future

（） I choose to receive the payment today.

（）I choose to receive the payment in the future.

If you select “I choose to receive the payment today”, then you will receive 100 dollars today.

If you select “I choose to receive the payment in the future,” then you will receive 120 dollars in 2 weeks.

Next

**How your earnings are determined**

After you finish making your decisions in all 24 rounds, one round will be randomly selected for payment. The amount and the timing of your payment will depend on the option you have chosen in this randomly selected round.

**How your earnings are paid**

Your earnings will be paid to you through wire transfer. If your payment date is in a few weeks, we will schedule the money to be wired to you on this future date. If your payment date is today, we will schedule the money to be wired to you today. The guaranteed $100 participation fee will be paid to you in cash right after the session.

Next

The experiment will start soon. If you have any questions, please raise your hand and the experimenter will come to assist you.

Next

(The experiment will start after clicking “Next” )

**A.2 Main task**

*****This is a sample round. The main task consists of 24 rounds.*****

Round 1

Please click “Next Page” to play the audio recording.

(The content of the audio recording is “I will receive 105 dollars in 4 weeks.” After Audio recording is played, the text below will be shown on screen.)

Please choose if you would like to receive the payment today or in the future

（）I choose to receive the payment today.

（）I choose to receive the payment in the future.

Next

**A.3 Post-experiment questionnaire**

1. What is your gender?

Male

Female

Not assigned

1. What is your blood type?

Type A

Type B

Type AB

Type O

Unknown

None of the above

1. Are you a local Taiwanese student?

Local Taiwanese student

Non-local Taiwanese student (e.g. overseas Chinese student, exchange student)

1. Are you an economics or business major?

Yes

No

1. How many economics courses have you taken?

0

1

2

3

4

5

More than 5

1. What is your year of study?

Year 1

Year 2

Year 3

Year 4

Year 5 or above

Graduate student

1. Would you please guess the purpose of the experiment?

**B. Instructions, main task, and exit survey (Original version)**

**B.1 實驗說明**

感謝您的參與。本場實驗包括主實驗項目以及實驗後問卷，總共所需時間約為30分鐘。除了保證的100元出席費之外，根據您所做的決定以及運氣，您的實驗報酬還包括了您在實驗中所賺取的報酬。請仔細閱讀以下實驗說明。

如果您在過程中有任何問題，請舉起手，實驗者會前往協助。現在請把您的手機與其他電子設備調為靜音，並維持靜音模式直到實驗結束。

在本實驗中，您的報酬將會以新台幣計算。

為確保無記名原則，您在實驗過程中的抉擇將只會連結至您的參與者代碼。雖然在實驗完成後，因會計作業需求我們會需要您提供姓名，但後續研究者無法連結您的姓名與參與者代碼，也就是說您在實驗中的抉擇仍是保密的。

Next

**決策實驗**

本決策項目共有24個回合，每個回合裡，您將在「今天得到100元」與「x週後得到y元」進行抉擇，其中x為正數，y的值大於100。也就是說，您是否願意等待幾週以獲得高於100元的報酬。請注意，每個回合的時點（x）與金額（y）都會不同，請您仔細閱讀與聆聽。

請戴上耳機，點選下一頁。

Next

**範例**

表單的頂端

在進入正式的回合之前，我們先給您一個例子。

請點擊播放語音

表單的頂端

註：這邊的金額和日期僅是範例，在真實的實驗中，每回合的金額和日期都會不同。

請選擇您要今天或未來的報酬:

- 我選擇今天的報酬

- 我選擇未來的報酬

如果您選擇今天的報酬，那代表您今天會得到100元。

如果您選擇未來的報酬，那代表您會在2週後得到120元。

Next

## 實驗報酬決定方式

當您完成本實驗24個回合的所有選擇後，電腦將隨機選出某個回合來兌現您的報酬。報酬金額以及獲得報酬的時間將會取決於這回合中您的抉擇。

## 付款方式

您的實驗報酬將會以匯款的方式支付。要是您收款的日期是數星期之後，匯款的日期也會隨之變動，而您得等到約定的時間之後才會收到匯款。要是您獲得報酬的日期是今天，那麼收到匯款金額的日期也會是今天。除此之外，保證的100元出席費將在實驗後直接以現金支付給您。

Next

表單的頂端

實驗即將開始，若有任何疑問，請舉起手，實驗者會前往協助。

Next

## B.2 主要決策項目

## 第1回合

**(由於各回合作答介面皆相同，故此處以第一回合為例)**

表單的頂端

請點擊播放語音

請選擇您要今天或未來的報酬:

- 我選擇今天的報酬

- 我選擇未來的報酬

Next

表單的底部

**B.3 實驗問卷**

表單的頂端

1.您的性別是:

- 男

- 女

- 不指定

2.您的血型是:

- A型

- B型

- AB型

- O型

- 未知

- 以上皆非

3.您是否為台灣本地生:

- 台灣本地生

- 非台灣本地生（例如僑生、外籍交換生學生）

4.您是否是經濟系或管理學院的學生:

- 是

- 否

5.您修過多少門經濟學的課程:

- 0門

- 1門

- 2門

- 3門

- 4門

- 5門

- 超過5門

6.您的系級是:

- 大學部1年級

- 大學部2年級

- 大學部3年級

- 大學部4年級

- 大學部5年級以上

- 碩士班學生

7.請您猜測本實驗的目的為何:

Next

表單的底部
